# The effects of a hirudin/liposome complex on a diabetic nephropathy rat model

**DOI:** 10.1186/s12906-019-2531-7

**Published:** 2019-06-06

**Authors:** Hongwu Wang, Huantian Cui, Lan Lin, Yue Ji, Qing Ni, Junchen Li, Jianli Pang, Gongyan Bing, Yuhong Bian

**Affiliations:** 1 Tianjin University of Traditional Chinese Medicine, No.10, Poyang Road, Jinghai District, Tianjin, 301617 China; 2 Guang’anmen Hospital, China Academy of Chinese Medicine Sciences, No.5, Beiji Pavilion, Xicheng District, Beijing, 100053 China; 3 The Second Affiliated Hospital of Guangxi University of Traditional Chinese Medicine, No.10 Hua Dong Road, Nanning, 530011 Guangxi region China; 4 The Affiliated East Hospital of Beijing University of Traditional Chinese Medicine, No.6, Zone 1, Fangxingyuan, Fengtai District, Beijing, 100078 China

**Keywords:** Diabetic nephropathy, Hirudin/liposome complex, Target therapy

## Abstract

**Background:**

Hirudin, an extract from *Hirudo spp.*, is an anticoagulant used to treat a variety of renal diseases, including diabetic nephropathy (DN). Currently, hirudin has to be used at high dosages to treat DN because it poorly targets the kidneys, although at high dosages it can have severe side effects. Developing a targeted drug delivery system for hirudin, then, could boost its positive therapeutic effects while lowering the risk of side effects. Liposomes have been demonstrated to have significant renal targeting potential, but here we show that a hirudin-loaded liposome is an effective delivery method for patients with DN.

**Method:**

In this study, we prepared a hirudin/liposome complex and tested its efficacy by injecting it into a rat model. We then compared the renal accumulation of hirudin between complex-injected rat models and rat models that received injections of hirudin alone. We also investigated the mechanisms behind the complex’s effects.

**Result:**

The hirudin/liposome complex increased the accumulation of hirudin in kidney tissues and relieved the renal injury in DN rat models. Moreover, the hirudin/liposome complex down-regulated the expression of TGF-β1 and VEGF in the kidneys.

**Conclusion:**

We demonstrated that a hirudin/liposome complex can have a significant positive effect on DN. The mechanism may be that the complex inhibits the expression of VEGF and TGF-β1.

## Background

Diabetic nephropathy (DN) is a common complication from diabetes. Approximately 25–50% of diabetes patients suffer from the condition [1], which causes a progressive loss of renal function. Poor renal function can trigger end-stage renal disease (ESRD), and ultimately lead to renal failure [1]. Currently, the pathogenesis of DN is unclear. Recent studies have shown the progression of DN is related to the dysfunction of vascular endothelial growth factor (VEGF) and transforming growth factor β-1(TGF-β1), which may contribute to the pathological changes of glomeruli and renal tubules [2, 3].

Traditional Chinese medicine (TCM) has shown promise in the treatment of DN. Shuxuetong injection can significantly improve the renal function in DN patients [4], while Huangkui Capsule, an extract from *Abelmoschus manihot* (L.), improves their microinflammatory status [5]. Bailing capsules relieve microinflammation and oxidative stress in patients with DN-induced chronic renal failure [6]. Finally, the Tang-Wei-Kang pills, made of extract from *Astragalus*, *hirudo,* and *Schisandra chinensis*, are known to have a positive effect on patients with DN [7, 8], up-regulating the expression of Matrix metallopeptidase 9 (MMP-9), while down-regulating the expression of TIMP metallopeptidase inhibitor 1 (TIMP-1) in the kidneys of DN rat models [8, 9]. However, nonspecific targeting is a disadvantage in the clinical use of TCM. Patients normally need long periods of treatment with TCM, and this can be expensive.

Hirudin, an extract from *Hirudo* spp., has been used as an anticoagulant in many renal diseases, including chronic renal failure, glomerulonephritis, and DN [10, 11]. Hirudin can be used clinically as an alternative anticogulant for heparin in hemodialysis [12]. It decreases urine albumin and improves hypercoagulable states in the kidneys of DN patients [13]. However, the targeting efficiency of hirudin is low, obligating that it be used in high doses. This is both wasteful and can result in significant side effects, such as hemorrhaging [14]. Enhancing the drug’s ability to accumulate in the kidneys, therefore, could increase hirudin’s therapeutic effects while decreasing the potential for harmful side effects at the same time.

Targeted therapy uses small (10-200 nm) pharmaceutical carriers, such as low molecular weight proteins, microspheres, micro-capsules, and liposomes to encapsulate drugs and deliver them to specific organs [15, 16]. Liposomes consist of phospholipid and quaternized cholesterol. They have high permeability, are widely used to deliver drugs to a target position [17, 18], and have high renal targeting potential. A recent study showed that the accumulation of doxorubicin in the kidneys of rats that received an injection of doxorubicin-loaded liposomes was significantly higher compared to rats that received an injection of doxorubicin alone [19].

Given the effects of hirudin on DN, and the renal targeting potential of liposomes, we hypothesized that a hirudin-loaded liposome could increase the delivery of hirudin to the kidneys. In this study, we prepared a hirudin/liposome complex and compared the renal targeting between the hirudin/liposome complex to that of hirudin alone in a rat model. We also investigated the possible mechanisms behind the effects of the hirudin/liposome complex.

## Methods

### Animals and reagents

The following were used in the course of our experiments: Male Sprague Dawley rats (Weitonglihua CO., LTD., Beijing, China); Hirudin (Kekang medical science CO., LTD., Nanning, China); distearoyl phosphatidylcholine (DSPC) (TC1, Shanghai, China); streptozotocin (STZ) (Sigma-Aldrich Inc., St. Louis, USA); Cr, BUN, and total urine protein test kit (Beihuakangtai CO., LTD., Beijing, China); Rabbit-anti-rat VEGF and TGF-β antibody (Biosynthesis Biotechnology Co., Beijing, China); PV-6001 immunostaining test kit (Zhongshanjinqiao biology science CO., LTD., Beijing, China); Hirudin Elisa test kit (American Diagnostica Inc., USA); extract total RNA kit, first-stand cDNA reverse transcription kit, polymerase chain reaction kit and primers (TianGen Biotechnology Co., Ltd., Beijing, China).

### Building the hirudin/liposome complex

First, the betainylated cholesterol (BC) was synthesized [20]. To prepare the liposome, we started with DSPC and BC at a molar ratio of 4:3 The two were mixed and dissolved in a solvent of chloroform and methanol (v/v, 4:1). The solution was then dried at 40 °C to obtain a thin lipid film, which was hydrated at 60 °C until totally hydrated, and then sonicated and extruded five times using a 200 nm filter to obtain the liposome.

To assemble the hirudin/liposome complex, the DSPC/BC lipid film were prepared using the same method above, after which 7 mL of lipid film was hydrated with 18 mL of phosphate-buffered saline (PBS) that contained hirudin (66 mg/mL) at 60 °C until totally hydrated. Finally, the hydrated lipid film and the hirudin mixture were sonicated and extruded five times using a 200-nm filter to obtain the hirudin/liposome complex. The morphology of the hirudin/liposome complex were characterized using transmission electron microscopy (TEM). The size and stability of hirudin/liposome complex were measured using dynamic light scatter (DLS) within 1 week.

### In vitro release study of hirudin/liposome complex

In vitro time-dependent hirudin release from the liposome was preformed by an α-phthaldialdehyde (OPA) assay after incubating the hirudin/liposome complex at 4 °C and 37 °C respectively. Hirudin/liposome complexes (2 mg) were dispersed in 2 mL PBS for 60 h. The release of hirudin was determined every 5 h based on the OPA method [20].

### Animals

Fifty male SD rats 8 weeks of age were housed at room temperature (23 ± 1 °C) with a 12 h light/dark cycle (lights on from 0600 to 1800). Food and water were available ad libitum. All experiments were carried out according to the institutional regulations and national criteria for animal experimentation.

### Investigation of the accumulation of hirudin in the kidneys

Twenty rats were randomly divided between a hirudin group and a hirudin/liposome group. The hirudin group received only a hirudin injection (1 mg/kg) through the tail vein, while the hirudin/liposome group received a hirudin/liposome complex injection (1.16 mg/kg, 1 mg/kg hirudin relatively) through the tail vein. After 6 h, all animals were sacrificed by cervical dislocation. The kidneys were removed and 0.1 g of the tissue was weighed and put into 900 μl of normal saline, followed by ultrasonic trituration and centrifuge at 3000 rpm for 15 min to obtain a homogenate of tissue. The supernatant was obtained to determine the kidney’s hirudin levels.

### Enzyme-linked immunosorbent assay (ELISA)

As shown previously, the levels of hirudin in the renal tissue homogenate were measured with a microplate reader using commercially available ELISA reagents, according to the manufacturer’s instructions [21]. The ELISA results were verified using intra- and inter-assay coefficient of variation (CV), which for the same renal tissue homogenate were 6.97 and 7.20% respectively.

### Hirudin/liposome treatment

Fifty rats were randomly divided into 5 groups: a control group, a model group, a liposome group, a hirudin group, and a hirudin/liposome group. The control group received normal feeding. For 6 weeks, the rats in the model, liposome, hirudin, and hirudin/liposome groups all received a high-fat / high-sugar diet (base feed 64%, lard oil 8%, egg yolk powder 10%, sucrose 18%, and sodium cholate 1%) to induce insulin resistance. This was followed by an intraperitoneal injection of STZ (35 mg/kg) to induce DN [22]. Afterwards, all the rats with DN received the high-fat diet daily. Additionally, the model group received an intraperitoneal injection of 0.5 ml of normal saline for 12 weeks. The liposome, hirudin, and hirudin/liposome groups all received an intraperitoneal injection with 0.5 ml liposome, hirudin (1 mg/kg), and the hirudin/liposome (1.16 mg/kg with 1 mg/kg hirudin), respectively, for 12 weeks.

### Blood glucose levels of rats

The rats’ blood glucose levels were measured every 2 weeks after the STZ injection with glucose test strips (ACCU-CHEK, China).

### Renal function

Twelve weeks after the STZ injection, urine was collected using a metabolic cage, and blood was obtained from the retrobulbar plexus for serum biochemistry analysis. Blood samples were centrifuged at 3000 rpm for 15 min, and serum was collected and kept at − 80 °C for analysis as described below. Serum creatine (Cr), blood urea nitrogen (BUN), and 24-h urine protein were tested on a plate reader using a reagent kit.

### Histology

After the blood was obtained, all animals were sacrificed by cervical dislocation. The kidneys were removed and fixed in formalin. They were then embedded in paraffin and cut into 5 μM sections. Sections were stained with haematoxylin and eosin (H&E).

### RNA isolation and real-time reverse transcription quantitative polymerase chain reaction (RT-PCR)

Total RNA was isolated from the rat kidneys using an RNA extraction kit. First, strand cDNA was synthesized from 1μg of the total RNA according to the manufacturer’s instructions. Quantitative reverse transcription PCR (qRT-PCR) was used to detect the expression of β-actin, TGF-β1, and VEGF, as previously described [23]. All samples were run in triplicate and detected by BIORAd iQ5. β-actin was used as a loading control. Quantification was undertaken using the 2^-△△^CT method [24]. The sequences of all primers are listed in Table 1.

**Table 1:** Primer sequences of target genes in the rat model

Genes	Primer sequence (5′-3′)
*β-actin*	Forward: ACC CGC GAG TAC AAC CTT CT
	Reverse: TCA GGG TCA GGA TGC CTC T
*TGF-β1*	Forward: CTT TGG ATG CCG CCT ATT GC
	Reverse: CCC CAG CAC AGA AGT TAG CA
*VEGF*	Forward: CCT GGC TTT ACT GCT GTA CCT
	Reverse: GCT GGT AGA CGT CCA TGA ACT

### Immunostaining

The levels of VEGF and TGF-β1 in the kidneys were assessed by immunostaining as previously described [25]. The data were analyzed with Image Pro Plus. The relative levels of VEGF and TGF-β1 were quantified and compared to the control group. Twenty randomly selected color video images of 512 × 512 pixels with a resolution of 0.4348 μm were recorded. The integrated optical density was observed based on the RGB color parameter. To control for non-specific staining of primary antibodies, negative control experiments were conducted using the omission of the primary antibodies and the preadsorbtion of primary antibodies with a more than 25 x molar excess of the protein (VEGF and TGF-β1) to be raised against the primary antibody. In addition, specific immunostaining patterns of VEGF and TGF-β1 in rat kidneys were used as positive controls.

### Statistical analysis

All data were analyzed using a one-way analysis of variance (ANOVA) followed by Newman–Keuls multiple-test comparison (mean ± SD). Statistical significance was determined at a *p*-value < 0.05. Analyses were done with SPSS version 20.0. Curve-fitting was carried out using GraphPad Prism5.

## Results

### Characterization of hirudin/liposome complex

**Fig. 1:**
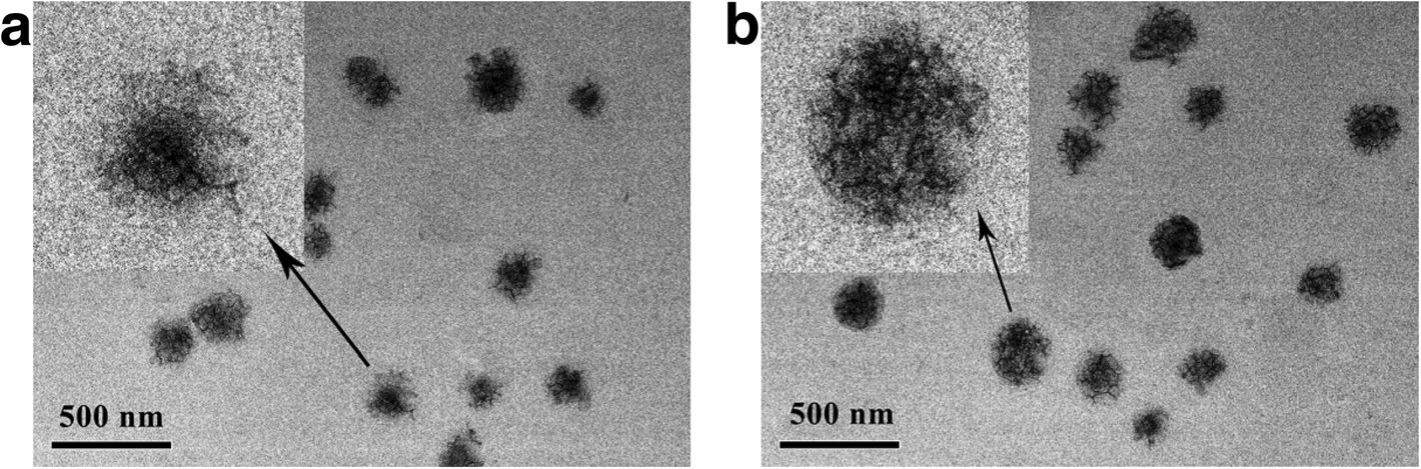
**a** TEM image of liposome; and **b**) the hirudin/liposome complex

**Fig. 2:**
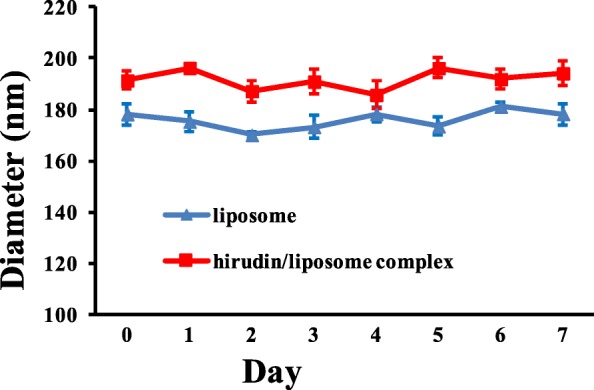
DLS spectra showing the particle size of the liposome and the hirudin/liposome complex did not change significantly within one week

The morphology of the liposome and hirudin/liposome complex was observed by TEM. As shown in Fig. 1, both the liposome and the hirudin/liposome complex were evenly distributed and had a uniform spherical shape, with an average size of ~ 180 nm and ~ 190 nm respectively. Dynamic light scattering (DLS) measurement showed the initial liposome particle size was 178.31 ± 4.18 nm and the initial particle size of the hirudin/liposome complex was 191.49 ± 3.67 nm (Table 2). No significant changes were observed in the size of the liposome or the hirudin/liposome complex within 1 week in PBS (Fig. 2), indicating it was stable.

**Table 2:** Size and Zeta potential of liposome and hirudin/liposome complex

	Diameter (nm)	Polydispersity	Zeta potential (mV)
Liposome	178.31 ± 4.18	0.16 ± 0.03	−16.15 ± 0.53
Hirudin/liposome complex	191.49 ± 3.67	0.14 ± 0.02	−15.82 ± 0.71

### In vitro hirudin release of hirudin/liposome complex

**Fig. 3:**
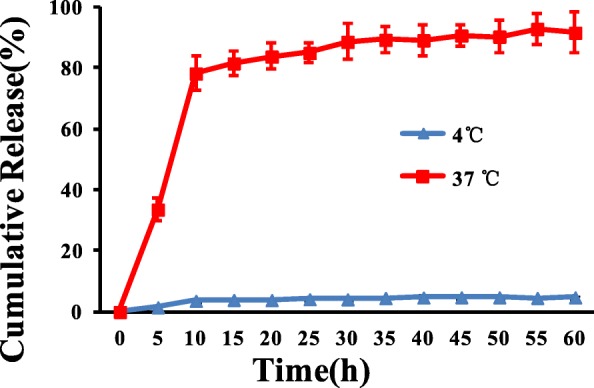
In vitro time-dependent hirudin release from hirudin/liposome complex at 4 °C and 37 °C respectively

To determine whether hirudin could be released from the hirudin/liposome complex, we investigated the release kinetics at different times. We further explored whether hirudin could be released from the hirudin/liposome complex spontaneously during storage, and compared the hirudin release at 4 °C and 37 °C, respectively. As shown in Fig. 3, hirudin was barely evident at 4 °C, with < 5% release, but the rate increased continuously in the first 10 h at 37 °C. Nearly 30% of total hirudin release was observed within the first 5 h, and ~ 80% release over 10 h. These results showed that the hirudin/liposome complex was stable at 4 °C and rapidly released hirudin at a standard physiological temperature.

### Accumulation of hirudin in kidney

**Fig. 4:**
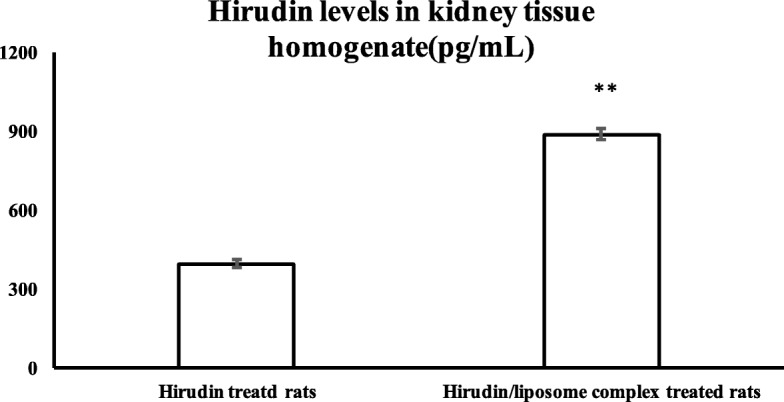
Six hours after injection of the hirudin/liposome complex, hirudin levels in the kidneys were significantly higher in hirudin/liposome complex-treated rats compared with the hirudin-treated rats. Hirudin treated rats (*n* = 10); Hirudin/liposome complex treated rats (*n* = 10). **:*P*<0.01 compared with hirudin-treated rats

Six hours after hirudin and the hirudin/liposome complex had been injected, the degree of hirudin accumulation in the kidneys was analyzed using ELISA. Hirudin levels were significantly higher in rats that received the hirudin/liposome complex injection compared to rats injected with hirudin alone (*P*<0.01, Fig. 4), indicating that the hirudin/liposome complex led to greater accumulation of hirudin in kidney tissues.

### Blood glucose in DN rat models

**Fig. 5:**
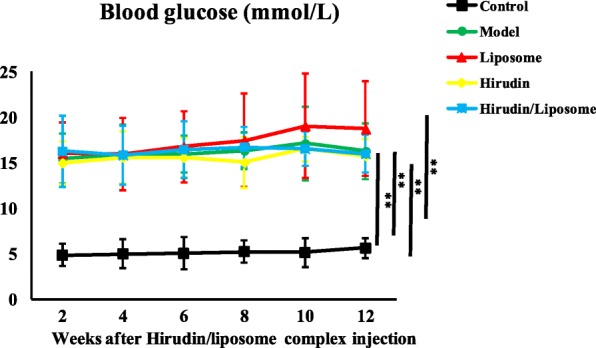
After STZ injection, levels of blood glucose in the Model, Liposome, Hirudin and the Hirudin/liposome complex groups were significantly higher compared with the Control group, although the hirudin/liposome complex treatment did not lead to a decrease in the blood glucose level in DN rat models. Control group (n = 10), model group (*n* = 7), liposome group (n = 7), hirudin group (*n* = 8), hirudin/liposome complex group (n = 7):*P*<0.05;*:*P*<0.01

Two weeks after the STZ and hirudin/liposome complex treatments, blood glucose levels had increased in the Model, Liposome, Hirudin, and Hirudin/liposome complex groups compared with the Control group (*P* < 0.01, respectively Fig. 5), indicating that rats had acquired DN. Twelve weeks after the STZ treatment, blood glucose levels were still higher in the four treatment groups compared to the Control group (*P* < 0.01, respectively Fig. 5), but there was no significant difference in the blood glucose levels among the Liposome, Hirudin and Hirudin/liposome complex, and Model groups (*P*>0.05, Fig. 5).

### Renal function in DN rat models

**Fig. 6:**
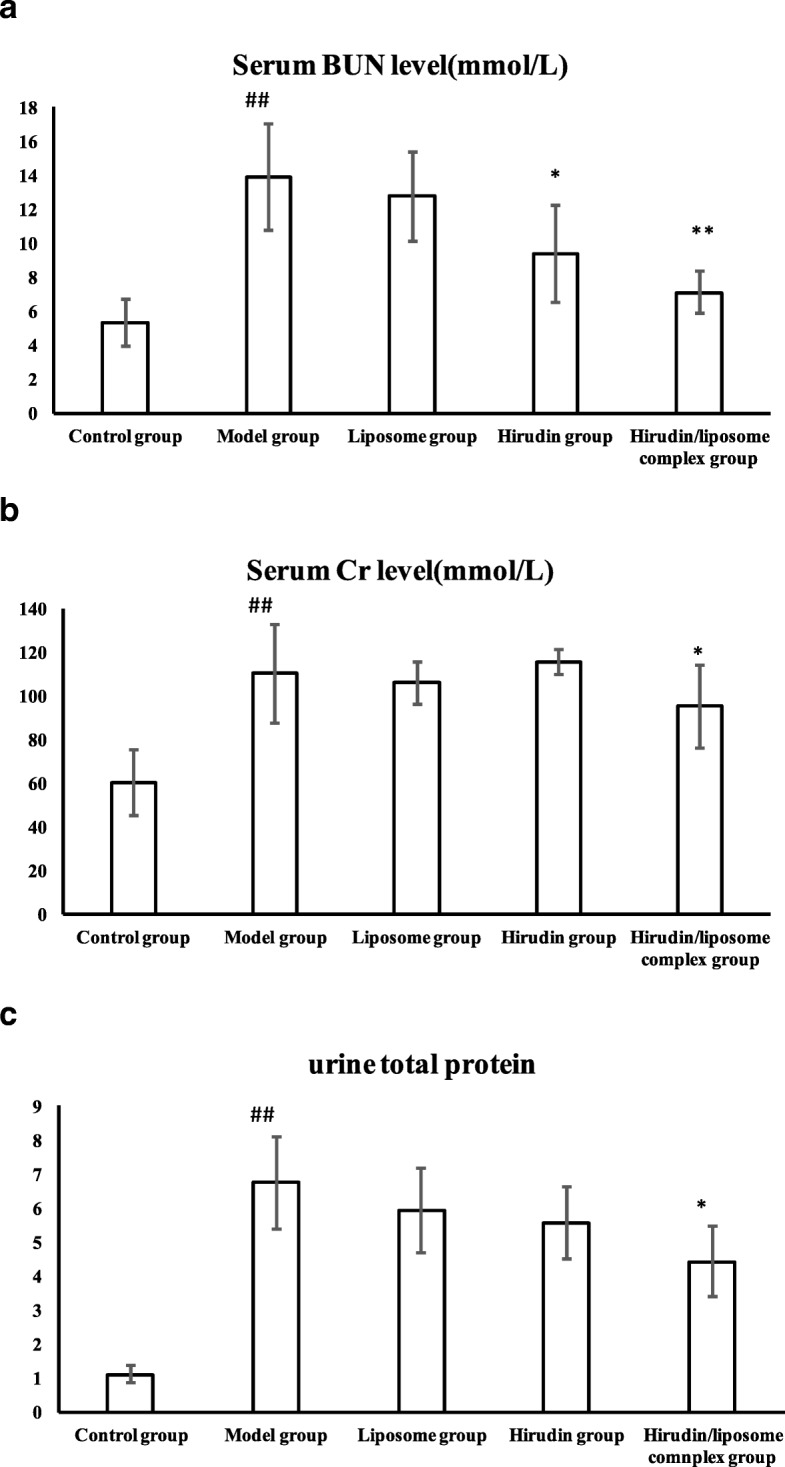
The hirudin/liposome complex treatment reduced the levels of serum BUN (**a**), Cr (**b**), and 24-h urine protein (**c**) in DN rat models. Control group (n = 10), Model group (*n* = 7), Liposome group (*n* = 7), Hirudin group (*n* = 8), Hirudin/liposome complex group (*n* = 7). ##:*P*<0.01 compared with the Control group; *:*P*<0.05 compared with the Model group;**:*P*<0.01 compared with the Model group

BUN and Cr levels were significantly higher in the Model group compared with the Control group (*P* < 0.05, Fig. 6a, b). However, BUN levels were significantly lower in both the Hirudin (*P* < 0.05, Fig. 6a) and the Hirudin/liposome complex groups (*P* < 0.01, Fig. 6a) compared to the Model group. Cr levels were lower as well in the Hirudin/liposome complex group compared to the Model group (*P* < 0.05, Fig. 6b). Levels of 24-h urine protein were higher in the Model group compared to the Control group (*P* < 0.01, Fig. 6c), but were lower in the Hirudin/liposome complex group compared to the Model group (*P* < 0.05, Fig. 6c).

### Pathological findings in DN rat models

**Fig. 7:**
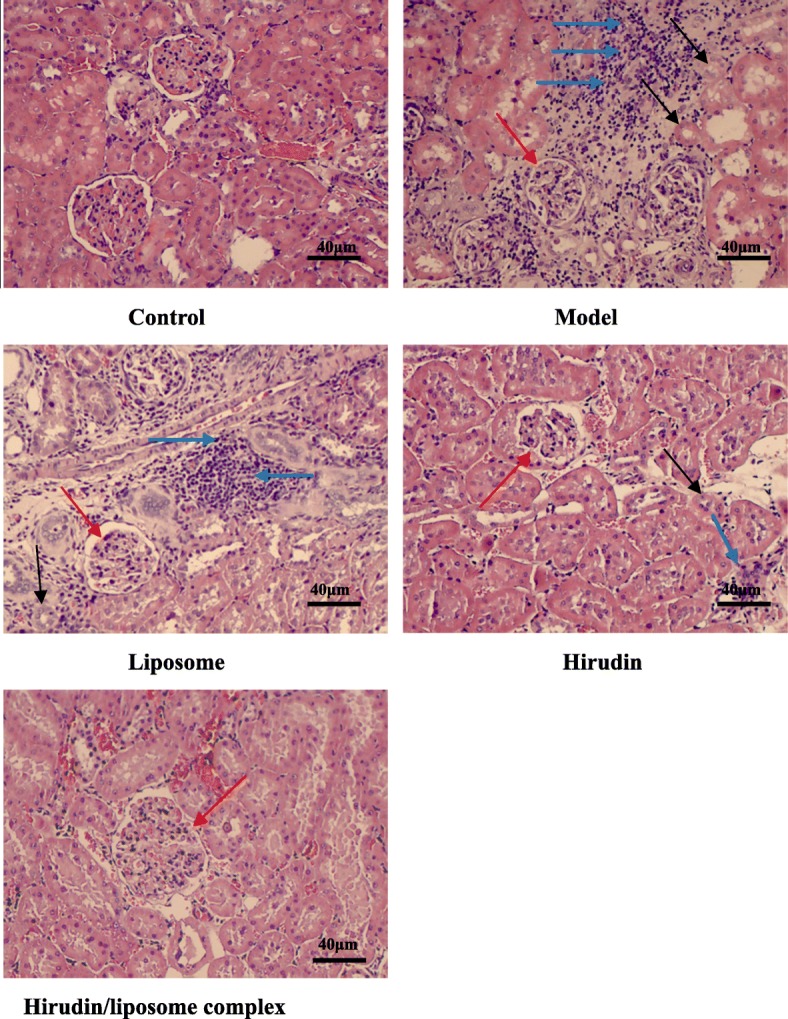
HE staining indicated that pathological changes including the hyperplasia of the glomerular mesangial matrix, tubular atrophying and infiltration of inflammatory cells could be noted in Model group. The hirudin/liposome complex treatment reduced the atrophying of kidney tubules and infiltration of inflammatory cells in the DN rat model. (Red arrows indicate hyperplasia of the glomerular mesangial matrix; black arrows indicate atrophying of kidney tubules; blue arrows indicate infiltration of inflammatory cells, 20x). Control group (*n* = 10), Model group (*n* = 7), Liposome group (*n* = 7), Hirudin group (*n* = 8), Hirudin/liposome complex group (*n* = 7)

Compared to the rats in the control group, rats in the Model group developed marked pathological changes in the glomerulus, including hyperplasia of the glomerular mesangial matrix. In addition, tubular atrophying and infiltration of inflammatory cells could also be noted in the model group. The infiltration of inflammatory cells was relieved in both the Hirudin group and the Hirudin/liposome complex group. The latter also showed a clear reduction in the atrophying of the kidney tubules (Fig. 7).

### *VEGF* and *TGF-β1* gene expression in kidney tissue

**Fig. 8:**
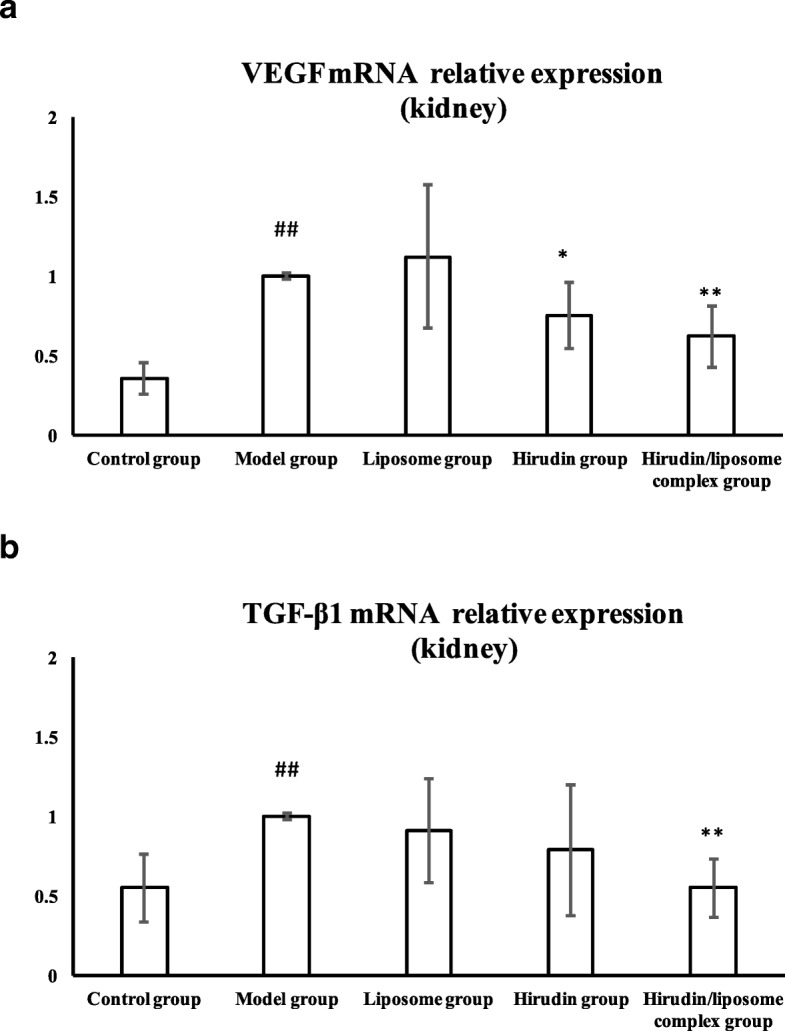
Gene expression of *VEGF* (**a**) and *TGF-β1* (**b**) in the kidneys was down-regulated in the Hirudin/liposome complex group compared with the Model group. Control group (*n* = 10), Model group (*n* = 7), Liposome group (*n* = 7), Hirudin group (*n* = 8), Hirudin/liposome complex group (*n* = 7). ##:*P*<0.01 compared with Control group; *:*P*<0.05 compared with Model group

We investigated the gene expression of *VEGF* and *TGF-β1* in the kidneys using qRT-PCR. The expression of both *VEGF* and *TGF-β1* in the kidneys was up-regulated in the Model group compared with the Control group (*P*<0.01, Fig. 8a, b), whereas *VEGF* expression was down-regulated in the Hirudin and Hirudin/liposome complex groups compared to the Model group (*P*<0.05 and *P*<0.01, respectively Fig. 8a) *TGF-β1* expression in the kidneys was down-regulated in the Hirudin/liposome group compared with the Model group (*P*<0.05, Fig. 8b).

### Immunostaining of VEGF and TGF-β1 in kidney tissue

**Fig. 9:**
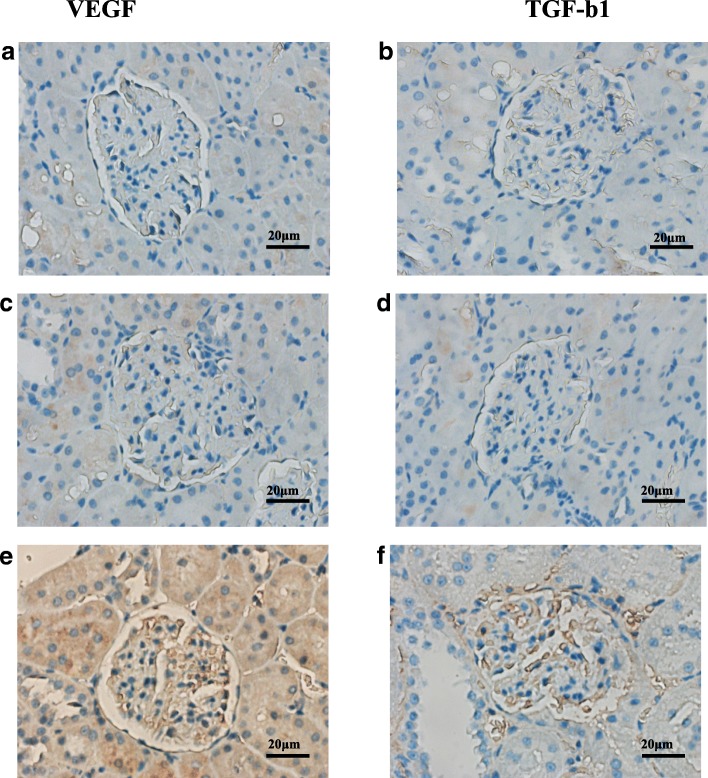
The negative controls of VEGF (**a**) and TGF-β1 (**b**) and pre-adsorbtion controls of VEGF (**c**) and TGF-β1 (**d**) showed a complete abolishment of specific signals for all antibodies. Positive contols for VEGF (**e**) and TGF-β1 (**f**) staining showed a apecific staining partterns in the vascular epithilial cells for VEGF and in the stroma cells for TGF-β1(40x)

**Fig. 10:**
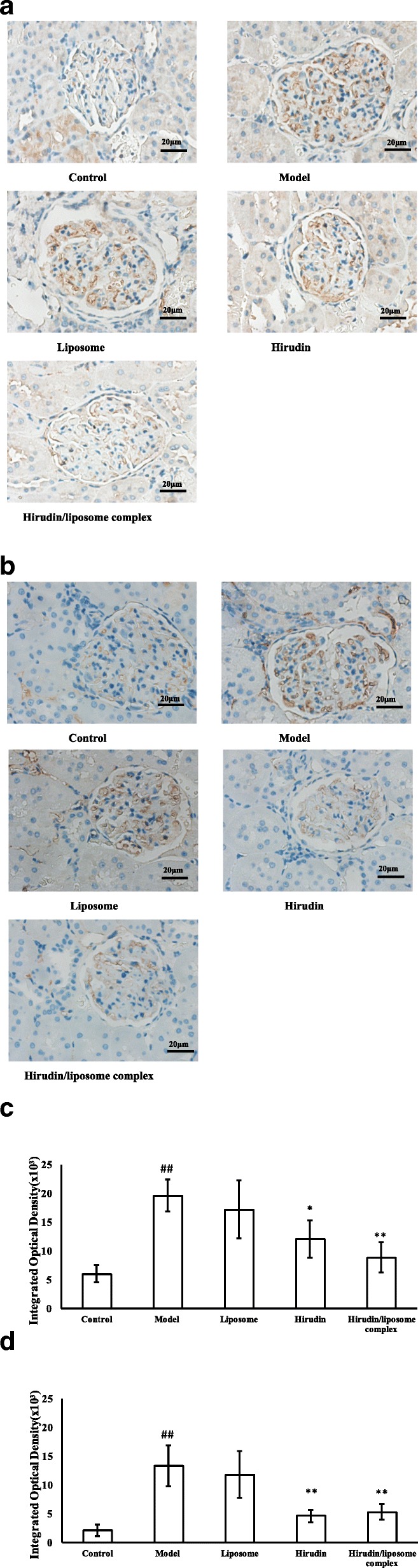
Immustaining indicated that VEGF levels in the epithelium of the glomerulus (**a**, **c**) and TGF-β1 (**b**, **d**) levels in the stroma of the glomerulus and kidney tubules were decreased in the Hirudin/liposome complex group compared with the Model group (40x). Control group (*n* = 10), Model group (*n* = 7), Liposome group (*n* = 7), Hirudin group (*n* = 8), Hirudin/liposome complex group (*n* = 7). ##:*P*<0.01 compared with Control group; *:*P*<0.05 compared with Model group;**:*P*<0.01 compared with Model group

The protein levels of VEGF and TGF-β1 in kidneys were measured using immunostaining. The positive, negative, and preadsorbtion controls of immunostaining were shown in Fig. 9. As shown in Fig. 9, VEGF was expressed in the epithelium of the glomerulus (Fig. 9e), and TGF-β1 was expressed in the stroma of the glomerulus and kidney tubules (Fig. 9f). Consistent with the qRT-PCR data, the levels of VEGF and TGF-β1 were higher in the Model group compared with the Control group (*P*<0.01, Fig. 10a-d). Moreover, VEGF levels decreased in the Hirudin group and the Hirudin/liposome complex group compared to the Model group (*P*<0.05 and *P*<0.01, Fig. 10a, c). The levels of TGF-β1 decreased in the Hirudin group and the Hirudin/liposome complex group compared with the Model group (*P*<0.01, Fig. 10b, d). There were no significant differences in renal VEGF and TGF-β1 expression in the Liposome group compared with the Model group (*P*>0.05, Fig. 10a-d).

## Discussion

In this study, we compared the accumulation of hirudin in the kidneys when it was injected by itself and when it was administered via a hirudin/liposome complex. Our results show that the degree of hirudin accumulation in the kidneys of rats that received the hirudin/liposome complex injection significantly increased, indicating a significant renal targeting effect of the complex.

Additionally, we established the animal model of DN through a high-fat and high-sugar diet and an injection of STZ. Rats in the model group exhibited severe renal injury, which was reflected in the increasing levels of blood glucose, Cr, BUN, and 24-h urine protein. Furthermore, pathology studies of the kidney showed hyperplasia of the glomerular mesangial matrix, atrophying of kidney tubules, and the infiltration of inflammatory cells. These results indicate that DN in rats went into a proteinuria stage. Results further showed the hirudin/liposome complex could relieve the renal injury in the DN rat model. However, neither hirudin nor the hirudin/liposome complex could influence blood glucose levels.

VEGF could contribute to the transformation of endothelial cells into a vascular loop and influence the glomerular filtration membrane system, which plays an important role in vascular formation. Abnormal expression of VEGF after kidney maturation is one of the mechanisms in proteinuria. The overproduction of VEGF could alter the permeability of the glomerulus and contribute to proteinuria [2]. This study demonstrated that inhibiting the expression of VEGF may be one function of the hirudin/liposome complex.

We also demonstrated that the hirudin/liposome complex could inhibit the expression of TGF-β1. Accumulation of the extracellular matrix is a cause of glomerulosclerosis and tubulointerstitial fibrosis in the pathological process of DN. TGF-β1 could also induce the adhesion and sedimentation of the intercellular matrix and inhibit the degradation of the cellular matrix, finally contribuing to tubulointerstitial fibrosis [3, 26]. Therefore, inhibiting the TGF-β1 could slow or even halt the progression of DN.

## Conclusion

Our study demonstrated that the hirudin/liposome complex has significant renal targeting effects and thus can have a positive treatment effect on DN. The mechanism of the hirudin/liposome complex may be that it inhibits the expression of VEGF and TGF-β1 in the kidneys.

## Data Availability

The authors declared the datasets used and/or analysed during the current study were available from the corresponding author on reasonable request.

## Abbreviations

BUN: Blood urea nitrogen
Cr: Creatinine
DN: Diabetic nephropathy
STZ: Streptozotocin
TCM: Traditional Chinese medicine
TGF-β1: Transforming growth factor β-1
TIMP-1: Tissue inhibitor of metalloproteinase-1
VEGF: Vascular endothelial growth factor

## References

[1] GM Pang Y, Yan PZ (2010). Clinic draft specification of traditional Chinese medicine about diabetic peripheral neuropathy. China J Tradit Chin Med Pharm.

[2] Robinson ES, Matulonis UA, Ivy P, Berlin ST, Tyburski K, Penson RT, Humphreys BD (2010). Rapid development of hypertension and proteinuria with cediranib, an oral vascular endothelial growth factor receptor inhibitor. Clin J Am Soc Nephrol.

[3] Liu G, Li J, Gao J (2011). TGDGBX inhibits transforming growth factor-β-1 triggered lung fibroblast-myofibroblast trans differentiation and collagen production in vitro. Acta Universitatis Medicinalis Anhui.

[4] Min XL, Lan LG (2008). Effect of Shu-Tong injection on diabetic nephropathy. Shaanxi J Tradit Chin Med.

[5] Gao ZT, Wang G (2011). Effect of Huangkui capsule on micro-inflammation in diabetes patients [J]. Chin J Integr Tradit West Nephrol.

[6] Huang L, Liao TT, Gong J, Luo WF (2014). The influence of bailing capsule on microinflammatory state in diabetic nephropathy with chronic renal failure. China Mod Med.

[7] Lin L, Ni Q, Liu XM (2008). Tang-Wei-Kang capsule reverse diabetic nephropathy in 132 patients. J Med Res.

[8] Lin L, Guo L (2003). Effect of TWK on expression of MMP −9 in renal cortex in Streptozotocin - induced diabetic rats. J Shanxi Coll Tradit Chin Med.

[9] Lin L, Qing Q, Liu XM (2003). Effects and mechanisms of tang-Wei-Kang capsule protecting kidney function in diabetes rat. China J Chin Materia Medica.

[10] Glusa E (1998). Pharmacology and therapeutic applications of hirudin, a new anticoagulant[J]. Kidney Int Suppl.

[11] Pöschel KA, Bucha E, Esslinger HU (2000). Pharmacodynamics and pharmacokinetics of polyethylene glycol-hirudin in patients with chronic renal failure.[J]. Kidney Int.

[12] Vanholder RC, Camez AA, Veys NM (1994). Recombinant hirudin: a specific thrombin inhibiting anticoagulant for hemodialysis.[J]. Kidney Int.

[13] Ying LI (2010). Clinical study on hirudin in diabetic nephropathy with umalb as the main manifestations and hypertension kidney disease[J]. Chin J Clin Ration Drug Use.

[14] Neuhaus KL, Von ER, Tebbe U (1994). Safety observations from the pilot phase of the randomized r-Hirudin for improvement of thrombolysis (HIT-III) study. A study of the Arbeitsgemeinschaft Leitender Kardiologischer Krankenhausärzte (ALKK)[J]. Circulation.

[15] Gilbert RE, Kelly DJ, Atkins RC (2001). Novel approaches to the treatment of progressive renal disease. Curr Opin Pharmacol.

[16] Meijer DKF, Molema G, Moolenaar F, Zeeuw DD, Swart PJ (1996). (Glyco)-protein drug carriers with an intrinsic therapeutic activity: the concept of dual targeting. J Control Release.

[17] Franssen EJF, Moolenaar F, Zeeuw DD, Meijer DKF (1993). Low molecular weight proteins as carriers for renal drug targeting: naproxen coupled to lysozyme via the spacer L-lactic acid. J Med Chem.

[18] Ji H, Dong K, Yan Z (2016). Bacterial hyaluronidase self-triggered prodrug release for chemo-Photothermal synergistic treatment of bacterial infection[J]. Small.

[19] Lee HJ, Ahn BN, Paik WH (1996). Inverse targeting of reticuloendothelial system-rich organs after intravenous adm inistration of adriamycin-loaded neutral proliposomes containing poloxamer407 to rats. Int J Pharm.

[20] Zhao Y, Dai X, Wei X (2018). Near-infrared light-activated thermosensitive liposomes as efficient agents for Photothermal and antibiotic synergistic therapy of bacterial biofilm[J]. ACS Appl Mater Interfaces.

[21] Song XH, Huhle G, Wang LC (2000). Quantitative determination of PEG-hirudin in human plasma using a competitive enzyme-linked immunosorbent assay.[J]. Thromb Res.

[22] Jiang-Ping GU, Zhao L, De-Lin LI (2007). Effects of Hirudo on level of Endothelin-1 in diabetic nephyropathy rats. Chin Tradit Patent Med.

[23] Citri A, Pang ZP, Sudhof TC (2012). Comprehensive qPCR profiling of gene expression in single neuronal cells[J]. Nat Protoc.

[24] Livak KJ, Schmittgen TD (2001). Analysis of relative gene expression data using real-time quantitative PCR and the 2^-△△^CT method. Methods.

[25] Daoussis D, Tsamandas AC, Liossis SNC (2012). B-cell depletion therapy in patients with diffuse systemic sclerosis associates with a significant decrease in PDGFR expression and activation in spindle-like cells in the skin[J]. Arthritis Res Ther.

[26] Meng XM, Nikolic-Paterson DJ, Lan HY (2016). TGF-β: the master regulator of fibrosis. Nat Rev Nephrol.

